# Medical Nutrition Education for Health, Not Harm: BMI, Weight Stigma, Eating Disorders, and Social Determinants of Health

**DOI:** 10.1007/s40670-024-02025-9

**Published:** 2024-04-02

**Authors:** Kearney T. W. Gunsalus, Jordan K. Mixon, Ellen M. House

**Affiliations:** 1 Department of Biochemistry and Molecular Biology, Augusta University/University of Georgia Medical Partnership, Athens, GA USA; 2 Augusta University/University of Georgia Medical Partnership, Athens, GA USA; 3 Department of Psychiatry and Health Behavior, Augusta University/University of Georgia Medical Partnership, Athens, GA USA

**Keywords:** Medical nutrition education, Weight stigma, Eating disorders, Health equity/social justice/social and structural determinants of health

## Abstract

Effective nutrition training is fundamental to medical education. Current training is inadequate and can cause harm to students and patients alike; it leaves physicians unprepared to counsel on nutrition, places undue focus on weight and body mass index (BMI), can exacerbate anti-obesity bias, and increase risk for development of eating disorders, while neglecting social determinants of health and communication skills. Physicians and educators hold positions of influence in society; what we say and how we say it matters. We propose actionable approaches to improve nutrition education to minimize harm and pursue evidence-based, effective, and equitable healthcare.

## Introduction

Nutrition is fundamental to health and disease prevention [1]; a strong foundation in nutritional knowledge is therefore essential to medical education [2]. The estimated health burden of nutrition-associated conditions is significant and continues to increase [3]; current approaches to address this situation are not working [4]. We need to do better.

Nutrition education for medical students is often minimal [5–7], contains inaccuracies, and can harm both students and their future patients. It reinforces misconceptions and patterns of behavior detrimental to the health of students and patients alike. It can lead doctors to give bad advice, exacerbate weight stigma, and influence the development of eating disorders, while the omission of social and cultural determinants of diet leads to impractical recommendations that can alienate patients. Provider biases against obesity and inaccurate nutrition information disproportionately affect already-disadvantaged populations; rectifying our nutrition education is essential to create just, equitable, and successful healthcare.

Care should be taken in how nutrition is taught as medical schools seek to improve their nutrition curricula. We propose an evidence-based approach to nutrition education designed to support improved patient outcomes. We identify problematic practices in nutrition education from the perspective of both learners and patients, including limited evidence-based approaches to nutrition education, improper emphasis on body mass index (BMI), reinforcement of weight stigma, omission of eating disorders, lack of recognition of social determinants of health, and inadequate communication skills coaching. Nutrition and lifestyle medicine curricula have made significant progress in these areas in recent years, yet additional work remains [8–25]. We make recommendations (Table 1) and propose specific, actionable alternatives to address failings in nutrition curricula (Tables 2 and 3).

**Table 1:** Summary of recommendations

Medical nutrition education should:
• Be grounded in scientific evidence, including the macro- and micronutrients found in various foods, their digestion, absorption, and metabolism/storage, and their roles in health and disease
• Refocus from body mass index (BMI) and weight to health, emphasizing health behaviors, objective metrics of cardiometabolic health, and that health transcends specific BMI or weight targets
• Strive to counteract, rather than perpetuate, weight stigma, which is harmful to students and patients alike
• Be mindful of eating disorders and the impact physician language and behavior can have on their development
• Emphasize the importance of social and structural determinants of health (SSDOH) and cultural humility in all contexts, including discussions of diet, weight, and health
• Prioritize effective communication by including motivational interviewing and communication skills training for these complex conversations

## Medical Nutrition Education Leaves Doctors Unprepared

There is widespread agreement that nutrition is an essential component of medical education and that the curricular time devoted to it is insufficient [6, 26–30]. Although “Biochemistry and Nutrition” comprise 14–25% of the questions on the USMLE Step 1 examination [31], the Liaison Committee on Medical Education (LCME) does not mandate the inclusion of nutrition in medical curricula [32]. In 1985, the National Academy of Sciences recommended that preclinical medical curricula include at least 25–30 h of nutrition instruction [33], yet by 2012–2013 medical schools averaged 14.3 h of nutrition education, a number that has been declining [5, 34].

More importantly, medical students do not feel prepared to offer nutrition advice to patients [6] and do not perceive that nutrition is a curricular priority, but believe nutrition counseling is an important physician role [35]. Physicians who receive more nutrition training provide counseling to more of their patients [36]. However, the majority of physicians report receiving insufficient training to address their patients’ nutritional needs [36–41], despite 72–95% feeling that it is a physician’s responsibility to discuss nutrition with patients [36, 37, 40, 42].

## Biochemical Basis of Nutrition

A lack of nutrition knowledge can harm health. To provide nutrition advice specific to a patient’s health needs, physicians must understand the physiological roles of macro- and micronutrients and the biochemistry underlying their metabolism [43, 44]. Unfortunately, medical nutrition education frequently fails to contextualize this information or provide opportunities to practice its clinical application. Management of some disease states rely heavily on nutrition and metabolism [45–50]; some patients control their epilepsy through a “keto diet” [51]. However, the biochemical basis of this diet is frequently misunderstood—it is the exclusion of carbohydrates, not inclusion of fat, that leads to ketogenesis—leading to confusion in research and recommendations.

Foods and food groups are not inherently “good” or “bad,” and we should avoid moralization (e.g., “good carbs,” “bad fats”) and oversimplifying their relationship to health (e.g., “heart-healthy fats”) (Table 2). Consumption of essential fatty acids is vital, yet dietary guidelines fail to acknowledge this [52]. They recommend consumption of whole grains [52, 53], which are an important source of micronutrients and fiber for most people. However, because people commonly conflate “dietary carbs” (carbohydrate-rich foods, e.g. grains) with “biochemical carbohydrates” (e.g., sucrose, amylose), these guidelines often lead to the misconception that dietary carbohydrates are necessary for life or health. Well-intentioned doctors can thus malign patients’ dietary choices, believing, for example, that it is dangerous for a child with type 1 diabetes to use a low-carb diet (A. Martin, personal communication), despite evidence that it can improve glycemic control [54, 55]. Gluten-free diets are becoming increasingly common, and can lead to micronutrient deficiencies, as gluten-free alternatives typically lack the fortification of wheat products [56]. Discussing foods’ biological effects fosters a neutral approach that is open to each individual’s nutritional needs. Instead of conveying this nuanced reality, nutrition education too often reinforces overly simplistic messages and rigid, “one-size-fits-all” nutritional rules. For most people, there are many “right” ways to eat for good health.

### Recommendation

Nutrition education should include the macro- and micronutrients found in various foods; their digestion, absorption, and metabolism/storage; and their roles in health and disease (Tables 1 and 2) [27, 43, 44, 57, 58].

**Table 2:** Discussing diet and recognizing an individual’s life situation: Problematic practices in medical nutrition and recommendations for improvement

**Avoid**	**Try this instead**	**Why?**
Moralization of foods and food groups as “good” or “bad.” Example: “heart healthy fats,” “good carbs,” “bad fats”	Discuss the macro- and micronutrients found in various foods, their digestion, absorption, and metabolism/storage, and the impacts of any one nutrient deficiency or excess	Understanding nutritional needs is essential to making plans or recommendations for patients’ health A knowledge of foods’ nutritional content can help our patients meet their macro- and micronutrient needs in a variety of ways
Shaming or promoting certain types of food Examples: • Claiming organic produce or other expensive food items are necessary for health • Shaming or dismissing canned foods due to sodium content • Recommending or endorsing fad diets	If there is evidence that a food item promotes health better than a cheaper counterpart, share such evidence but acknowledge that not everyone has access to such foods Recognize the importance of shelf-stable products for certain individuals. Suggest strategies such as draining and rinsing canned foods to reduce sodium content Avoid promoting fad diets	Nutrition counseling must work within an individual’s life. If our guidance prioritizes perfection and shames what is accessible, we lose patients’ trust in our understanding of their circumstances and may cause patients to feel that healthy eating is not feasible We should promote health by maximizing the nutritional value available to any given patient Fad diets are usually not supported by robust evidence, may promote short-term, unsustainable changes, and may cause harm
Assuming everyone has equal access to and knowledge of how to prepare healthy meals Examples: • Ignoring the existence of food deserts, the ubiquity of ultraprocessed foods, and accessibility challenges many individuals face • Assuming patients have available cooking appliances and/or the time and ability to prepare their own meals	Proactively seek education on the role of social determinants of health in nutrition and disease Routinely discuss nutritional knowledge and current barriers to health with patients Prioritize practical and affordable nutrition approaches for patients Examples: • “What’s your understanding of how food choices can affect your health?” • “What gets in the way of eating a diet you consider to be ‘healthy’?” • Familiarize students with resources (e.g. local food programs, myplate.gov recipes) to provide actionable approaches	If a patient’s nutrition is harming their health, you must understand the barriers they face *before* providing guidance Sometimes our patients need education, other times being pointed towards a resource (i.e., food banks with fresh fruits and vegetables), and at other points directions on food preparation. Providing a “one-size-fits-all” approach to nutrition education can alienate patients and fall short of addressing their needs
Reducing food to its biological functions to the exclusion of the social and cultural aspects of eating Example: Marketing slogans like the USDA’s “Make every bite count” [52]	Discuss the environments and communities in which our patients eat Examples: • “Who does the cooking in your house?” • “Who do you eat with?” • “Outside of nutrition, when or why else do you find yourself eating?” Recognize the social and connecting nature of eating within a person’s life and community Examples: • Leaving space for consumption of celebratory foods such as cake at a wedding or birthday • Increased risk for malnutrition in disconnected older adults	Feeding behaviors serve various functions outside of nutrition for individuals and cultures. A deeper understanding of the why, with whom, and when people eat can help physicians support patients in their values and goals

## Refocusing Nutrition Education from BMI and Weight to Health

BMI is widely used in healthcare and taught in medical education as an indicator of an individual’s health [59]. Yet the relationship between BMI and health status is not simple, as recognized by major organizations [48, 60, 61]*.* BMI is not an accurate indicator of body composition (fast, bone, and muscle mass) [60]. Using BMI as the main indicator of cardiometabolic health misclassifies a large percentage of American adults: almost 50% of overweight individuals, 29% of people with class 1 obesity, and 16% of those with class 2 or 3 obesity are metabolically healthy using the parameters of blood pressure, triglycerides, cholesterol, glucose, insulin resistance, and C-reactive protein [62]. Meanwhile, 30% of people whose BMI is in the “healthy” range are cardiometabolically unhealthy when assessed by these same parameters [62]. While BMIs in the obese range are correlated with negative health outcomes (e.g., cardiovascular disease, diabetes), cardiometabolic health is much more strongly predictive of overall health than BMI (Table 3) [63, 64].

Furthermore, it is not clear that weight, in and of itself, is a readily modifiable risk factor (Table 3). Most people struggle to maintain weight loss over the long term, and many cycle up and down in weight [65, 66], which has been found to be more harmful than consistently maintaining a higher weight [59, 67, 68]. Additionally, weight is not a behavior, and thus is not subject to behavioral modification counseling [69]. Actionable items such as active minutes per week, number of sugary drinks consumed, and mobility, or objective measures such as blood pressure, pulmonary function tests, or hemoglobin A1c, can be monitored and improvements achieved independent of weight change [4]. Many of the health benefits of lifestyle modifications, such as improved blood pressure, blood glucose, and lipid profile, are commonly ascribed to weight loss, yet they often occur independent of weight loss [59, 64].

**Table 3:** Discussing weight and body mass index (BMI): problematic practices in medical nutrition and recommendations for improvement

**Avoid**	**Try this instead**	**Why?**
Using BMI categories (healthy, overweight, and obese) as a measure of health Example: Assuming cardiometabolic health because of a normal range BMI	Focus on lifestyle, diet, physical activity, and objective measures of health as better predictors of health outcomes	BMI can be a useful population-level tool, but on an individual level, focusing on this number can bias clinicians and often misses health markers with more utility and predictive value It is not clear if increased weight causes, or is simply correlated with, adverse outcomes
Focusing on weight as a modifiable risk factor in an individual’s health Examples: • “You need to lose weight to be healthy and control your diabetes.” • American Heart Association’s blanket recommendation to “lose weight” [73]	Use SMART goals to help patients adopt sustainable health practices, focusing on objective measures that are clearly linked to health outcomes: • Time spent physically active • Number of sugary drinks consumed • Blood pressure • Hemoglobin A1c • Lipid profile • Pulmonary function tests	Weight is less modifiable than many presume, and weight cycling causes adverse health outcomes Weight is not a modifiable behavior Many health benefits of lifestyle modifications occur independent of weight loss If weight is the primary endpoint and does not change, this can lead to loss of motivation for behavior change
Overlooking risks of underweight Example: American Heart Association’s “Definitions of Poor, Intermediate, and Ideal Cardiovascular Health” fails to mention BMIs < 18.5 [73]	Recognize and address health risks at all weights, including those individuals with low BMIs	Being underweight carries significant health risks despite being more socially and medically acceptable within Western culture The health risks of being underweight are often underappreciated within clinical interactions
Presuming presence, absence or severity of an eating disorder based on BMI Example: “This person cannot have an eating disorder because their BMI is normal.”	Be aware of and screen for eating disorders in patients of all weights when discussing nutrition, weight, and lifestyle	Eating disorder behaviors can be present in those with normal or elevated BMIs and carry significant associated health risks. BMI-based assumptions about eating disorder presence cause harm and create barriers to treatment
Perpetuating eating disorder thoughts and behaviors Examples: • “You look great, have you lost weight?” • “You should cut out all carbs.” • “I’m having a *cheat* day.” • Silent curriculum emphasizing weight as “bad” and weight loss as the goal for health	Diet culture can be pervasive and addressing this explicitly is essential in discussions about health Prioritize communication and education that emphasizes unbiased markers of health Communicate to patients that steps towards health can be accomplished at any weight	Focusing on weight loss can cause or exacerbate eating disorder cognitions and behaviors Physicians and educators have a privileged place in society and serve as role models to students, patients, and the public; the way we discuss nutrition and weight has ripple effects Modeling healthy approaches to nutrition, weight, and exercise is protective and health promoting for all
Reinforcing fear or ignoring harm regarding medication effects on weight when treating other health conditions Examples: • Overemphasizing risk of weight gain when prescribing oral contraceptives or selective serotonin reuptake inhibitors • Underplaying risk of cardiometabolic effects of antipsychotics	For all medications and treatment plans, become aware of the likely side effects, their rates, mitigating factors, and cultural discussions that occur outside of the exam room Provide a chance for patients to discuss their concerns and be aware of evidence to be able to counsel appropriately without bias or reactivity	Patient and provider bias regarding weight gain from medication can pervade interactions over health for many medical conditions. For many common medications, the Internet and cultural conversations, particularly around weight gain, will affect adherence and provider use, which should be addressed in an evidence-based manner within clinical interactions
Using shame and fear tactics as a motivating strategy for behavior change Example: “Your weight is killing you.”	Prioritize communication and education that emphasizes patient autonomy, values, and health • “What concerns do you have about your health?” • “What are your thoughts about your weight?” • “What goals do you have for your health?” • Ensure adequate practice in motivational interviewing during medical training	Using shame or fear can be counterproductive in moving patients towards a better state of health Motivational interviewing which emphasizes a patient’s values, motivations, skills, and autonomy, has the greatest evidence base in fostering behavior change

Emphasizing healthy behaviors instead of weight encourages positive health outcomes for all patients [4, 64, 70]. This also reduces blame and shame; patients may feel at fault if weight loss is not seen, which can lead to learned helplessness and cessation of behavior change despite health improvements (Table 3) [4, 70]. Recommendations support the use of a SMART (specific, measurable, achievable, relevant, and time-bound) goal framework to encourage patients to establish and maintain new behaviors regardless of weight changes [71].

Medical education, practice, and research have been closely entangled with cultural concepts of healthy body size [48, 72]. Even reputable sources downplay the risks of being underweight (Table 3) [73]. While the extreme ends of the BMI scale are correlated with negative health outcomes, nuances that students and physicians struggle to integrate into clinical decision-making include that this is less true of overweight and class I obesity [69, 74–77], that the association between higher BMIs and morbidity/mortality is significantly attenuated by health behaviors [78–80], and that correlation does not demonstrate causation [69, 77]. Some of this misapprehension reflects students carrying forward their prior beliefs, and some is taught. Case-based learning often includes the patient’s BMI, but not pertinent factors such as body composition, fat distribution, or mobility; this teaches students to rely on BMI to the exclusion of those factors. Many students focus on this measurement as an indicator of health, rather than considering objective measures of cardiometabolic health or information about the patient’s diet, exercise habits, health history, and medication list. In our experience, nutrition education provided to medical students typically emphasizes weight loss, ignoring the harms of weight cycling [81, 82] and the many studies demonstrating that a low BMI is associated with a higher mortality rate than a high BMI [76, 83].

### Recommendation

Because the relationship between BMI and health is complex, nutrition education should stress the problematic nature of BMI as a marker of individual health and convey that health transcends specific BMI or weight targets (Tables 1 and 3) [48, 65, 69, 77].

## Weight Stigma

Weight stigma permeates healthcare and harms patients; it is found in systemic and institutional approaches to patients as well as interpersonal interactions [84, 85]. Physicians demonstrate similar levels of weight bias [86] and registered dieticians higher levels [87] compared to the general population. This stigma commonly associates obesity with moral failures, laziness, and gluttony, overlooking the many biologic and systemic factors that intersect with weight [4, 65, 84]. Weight stigma is harmful to medical students [88], and can be reinforced by medical education [89]. Provider and internalized anti-obesity bias negatively impact patient care and patient health outcomes in several ways, thus contributing to the development of the very diseases weight loss recommendations are intended to prevent [4, 90].

Weight bias leads physicians to provide less empathic support and a lower standard of care to patients of higher weights [4, 65, 84]. Doctors are more likely to view overweight patients as non-compliant and attribute symptoms to a patient’s weight [65, 84, 91]. Rather than conduct the same testing and make the same diagnoses they would for normal-weight patients [4, 84], physicians advise weight loss, which harms health by encouraging weight cycling and contributing to internalized weight bias by implying patients are lazy and weak-willed [59, 69]. Higher-weight patients are less likely to be screened for problematic feeding behaviors or eating disorders, which carry significant health risks [92].

Experiencing weight stigma negatively impacts patients’ health behaviors [93, 94]. Anti-obesity bias experienced within the physician–patient relationship leads patients to delay seeking healthcare [4, 65, 84, 91]. Perpetuating weight stigma does not motivate patients to make “positive” changes; it pushes them into unhealthy coping mechanisms with significant long-term health risks, including increased consumption of alcohol, high-fat, high-sugar, and high-calorie foods, and decreased sleep quality and physical activity [65, 66, 93].

Weight stigma has adverse physiological effects; it causes chronic stress [90], as indicated by higher levels of C-reactive protein, cortisol, and oxidative stress, which contribute to negative health outcomes such as high blood pressure and diabetes [90, 93]. It has a myriad of negative effects on mental health [90, 95]. It is also predictive of weight gain, risk of obese BMI, and mortality—independent of baseline BMI [4, 90].

### Recommendation

Medical education should seek to end weight stigma in healthcare [48, 50, 65, 69, 77, 96–98]. Medical education and communication with patients should be centered on behaviors and objective measures that are clearly linked to health outcomes, as this models a healthy approach to nutrition, weight, and physical activity (Table 1).

## Eating Disorders

The explicit and implicit curricular focus on weight as a primary marker of health creates a specific lens for our learners that (1) trains them to under-recognize and inadvertently facilitate eating disorders among their future patients and (2) risks development or perpetuation of eating disorders among our trainees.

Eating disorders have one of the highest mortality rates of all mental illnesses, and they are associated with significant cost, morbidity, and greatly reduced quality of life [99, 100]. Eating disorders are seen at higher rates than type 2 diabetes in 15–24 year olds [101], but are rarely addressed or considered in medical nutrition education [102]. It is estimated that 14–20% of Americans will have had an eating disorder by age 40 [103]. This may not include the many individuals with subclinical symptoms that nonetheless negatively impact health, or those who go undiagnosed due to lack of access, stigma, or “normal” or high BMIs.

Medical education can shape recognition of and attitudes towards individuals with disordered eating. Physicians should not assume the presence, absence, or severity of an eating disorder based on an individual’s body type or weight (Table 3) [92, 104]. Overweight individuals are often told to lose weight without assessment for eating disorder risk, making it more likely that at-risk health behaviors (e.g., restriction, purging, over-exercise) will go unrecognized. This impairs their ability to access and receive appropriate treatment [98]. Learners should be taught that patients of all weights may suffer the negative physical and mental health outcomes of eating disorder behaviors and cognitions [105]. Nutrition counseling must always be done with an awareness and consideration of eating disorders, regardless of BMI. Trainees need to learn how to provide nutrition counseling that does not encourage risky eating behaviors or impair eating disorder treatment [92, 98].

When nutrition education contains anti-obesity messaging, it can cause harm by contributing to the onset or perpetuation of eating disorder behavior among patients and students [98, 106–108]. In one treatment center, 18% of patients affirmed that anti-obesity messaging contributed to the onset of their disorder, and these patients presented with more severe symptoms [107]. Educational curricula were the most common source of such messaging, and healthcare providers were a common source of weight stigma [107].

Medical nutritional curricula can impact the health of our trainees [106]. Globally, ~ 10% of medical students are at high risk for developing an eating disorder [109]. Recent studies of medical students in Lebanon, Pakistan, and China found eating disorder risk rates from 2 to 18% [110–112]. Nearly 1 in 6 American medical students (16%) is thought to be at high risk, although this estimate is based on data from the 1980s and 1990s [109]. While data on current prevalence are needed, a significant percentage of our trainees either have, or are at risk for, eating disorders.

Increasing an individual’s nutritional knowledge can lead to dietary improvements, such as increased consumption of fruits, vegetables, and fiber [7, 25, 113, 114], but elevated awareness can also be problematic. Nutrition education can foster weight dissatisfaction and encourage behaviors that can increase risk for disordered eating [106]. Further research is needed to examine how nutrition education impacts the feeding behaviors of medical students and their patients.

### Recommendation

Medical education should acknowledge eating disorders and the impact physician language and behavior can have on their development [92, 98, 105, 115], thereby protecting both patients and students (Tables 1 and 3).

## Social and Structural Determinants of Health

While adequate nutrition is essential for life, we must remember that food is also culturally and socially important. People connect with each other over meals and eat special foods in celebration or mourning. We encourage educators and physicians to recognize that eating cake at a wedding or dates to break the fast of Ramadan count for quality of life.

Effective nutrition education—to students and patients alike—must recognize the importance of both culture and social and structural determinants of health (SSDOH) [27, 29, 48, 116, 117]. Unfortunately, medical education frequently assumes that everyone has equal access to food and the knowledge and ability to prepare healthy meals, and overlooks common barriers to healthy eating such as food deserts, cost, availability of fresh vs. ultra-processed foods, and lack of cooking appliances (Table 2). We should not shame food that is not fresh, or dismiss canned foods due to their sodium content. Instead, we should make suggestions for how to modify such foods, as they may be all that is available to some families. Similarly, claims that organic produce is necessary for health fail to acknowledge that these foods come at an inaccessible price tag for many [29] and that the organic designation is not relevant to the nutrition content of the product (Table 2) [118, 119].

### Recommendation

Effective nutritional counseling starts with cultural humility and understanding a patient’s circumstances and needs to then offer effective suggestions that use or adapt what is available [27, 29, 43, 48, 117, 120]. Meeting a patient where they are can avoid creating distrust or a sense of futility and instead support people in making positive lifestyle changes. We recommend moving away from valuing food only for its nutrient content, and instead acknowledge the complex role that food plays in any individual’s life and culture (Tables 1 and 2) [30, 52, 121].

## How We Say It: Language and Effective Communication

The words [70, 122] and communication strategies [123] physicians use impact patient outcomes. Students and physicians must be taught that evidence does not support using shame to coerce individuals into behavior change; rather, it furthers discrimination, is counterproductive, and can cause harm (Table 3) [48, 65, 101]. Research clearly and consistently indicates that physicians should foster behavior change through motivational interviewing, where patients’ autonomy, values, and goals are primary [124]. The tenets of motivational interviewing—compassion, acceptance, evocation, and collaboration—shield against the shame and moralizing so often present in discussions of weight and nutrition. Medical students report wanting help learning how to talk to patients about weight; we suggest instead that students need to learn to talk constructively with patients about their goals, behaviors, and health.

### Recommendation

Nutrition curricula should include motivational interviewing and communication skills training to best serve future patients in these complex conversations (Tables 1 and 3) [2, 27, 43, 48, 116, 120, 124].

## Conclusion

Medical education is not adequately equipping students to care for their future patients’ nutritional needs. When education perpetuates weight stigma and encourages disordered eating habits, it hurts our students, their future patients, and the healthcare system as whole.

Effective medical education can instead help end this cycle and improve health for all. As medical educators work to improve nutrition education, we should be mindful of the challenges patients face, from the systemic to the interpersonal, and what students absorb from our explicit curriculum and faculty modeling. Nutrition education should be grounded in basic science, informed by robust and current evidence about the role of nutrition in health and disease, cognizant of social determinants of health, and emphasize communication skills to foster sustained, healthy behavior changes. There are many institutions and passionate educators committed to change, and over the last decade many nutrition and lifestyle medicine curricula have addressed these issues; there is still significant progress to be made in multiple areas. Making these changes in medical education can be challenging; nutrition touches every aspect of integrated medical curricula, may benefit from interprofessional education [125], and requires collaboration among faculty. This could be facilitated by future research to build expert consensus in the field. Medical educators want to help students develop into empathic, effective physicians; we hope our recommendations can help equip students with the knowledge and skills to discuss nutrition, weight, and health.
